# Sodium channel *SCN8A* (Na_v_1.6): properties and *de novo* mutations in epileptic encephalopathy and intellectual disability

**DOI:** 10.3389/fgene.2013.00213

**Published:** 2013-10-28

**Authors:** Janelle E. O'Brien, Miriam H. Meisler

**Affiliations:** Department of Human Genetics, University of MichiganAnn Arbor, MI, USA

**Keywords:** voltage-gated sodium channels, epilepsy, intellectual disability, *SCN8A*, Na_v_1.6, neurogenetics, genetics, exomes

## Abstract

The sodium channel Na_v_1.6, encoded by the gene *SCN8A*, is one of the major voltage-gated channels in human brain. The sequences of sodium channels have been highly conserved during evolution, and minor changes in biophysical properties can have a major impact *in vivo*. Insight into the role of Na_v_1.6 has come from analysis of spontaneous and induced mutations of mouse *Scn8a* during the past 18 years. Only within the past year has the role of *SCN8A* in human disease become apparent from whole exome and genome sequences of patients with sporadic disease. Unique features of Na_v_1.6 include its contribution to persistent current, resurgent current, repetitive neuronal firing, and subcellular localization at the axon initial segment (AIS) and nodes of Ranvier. Loss of Na_v_1.6 activity results in reduced neuronal excitability, while gain-of-function mutations can increase neuronal excitability. Mouse *Scn8a (med*) mutants exhibit movement disorders including ataxia, tremor and dystonia. Thus far, more than ten human *de novo* mutations have been identified in patients with two types of disorders, epileptic encephalopathy and intellectual disability. We review these human mutations as well as the unique features of Na_v_1.6 that contribute to its role in determining neuronal excitability i*n vivo*. A supplemental figure illustrating the positions of amino acid residues within the four domains and 24 transmembrane segments of Na_v_1.6 is provided to facilitate the location of novel mutations within the channel protein.

## Introduction

*SCN8A* encodes one of the major voltage-gated sodium channels that regulate the initiation and propagation of action potentials in the nervous system. The sodium channel transmembrane proteins were first purified 30 years ago (Hartshorne and Catterall, 1981; Tamkun and Catterall, 1981) and cDNA clones were isolated shortly thereafter (Noda et al., 1986). The *Scn8a* gene, encoding the sodium channel Na_v_1.6, was identified in 1995 by positional cloning of the mouse neurological mutant *motor endplate disease* (med) (Burgess et al., 1995) and by isolation of a novel sodium channel cDNA from rat brain (Schaller et al., 1995). *SCN8A* is a member of the gene family comprised of nine evolutionarily related sodium channels with specific roles in neurons and in skeletal muscle and cardiac muscle (Lopreato et al., 2001; Meisler and Kearney, 2005; Meisler et al., 2010; Zakon et al., 2011; Zakon, 2012).

Human *SCN8A* was mapped to chromosome 12q13 in 1998 (Plummer et al., 1998). The role of *SCN8A* in human disease was initially investigated by screening for mutations in families segregating inherited disorders such as ataxia, dystonia, and tremor (Trudeau et al., 2006; Sharkey et al., 2009a). These analyses identified only one family with an inherited mutation of *SCN8A* (Trudeau et al., 2006). Recently, the ability to sequence the entire exome or genome from an individual patient has made it possible to identification of *de novo* mutations in patients who do not have a family history of disease (Bamshad et al., 2011; Doherty and Bamshad, 2012; Need et al., 2012; Rauch et al., 2012). Using this technology, more than ten mutations of *SCN8A* have been described during the past year, in patients with epileptic encephalopathy and intellectual disability. This rapid progress indicates that mutations of *SCN8A* are a previously unrecognized cause of these and possibly other neurological disorders. Here we describe the recently discovered patient mutations and review the unique features of Na_v_1.6 as a framework for understanding the pathological consequences of human mutations.

## Mutations of *SCN8A* in patients with epileptic encephalopathy

**Figure 1:**
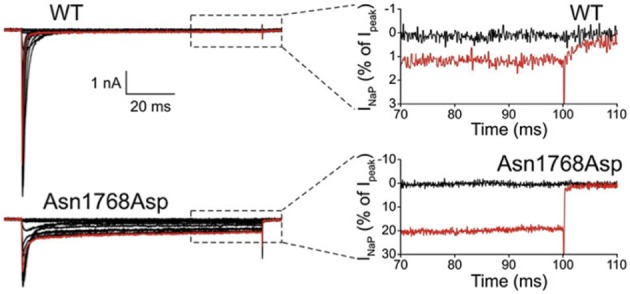
**Increased persistent current in *SCN8A*-p.Asn1768Asp mutant channel.** Wildtype and mutant cDNAs were transiently transfected into the neuronal cell line ND7/23. At 100 ms after induction of an action potential, cells expressing the mutant cDNA had 20% persistent current compared with 1% in the wildtype. Cells were held at −120 mV, and a family of step depolarizations (−80 to +60 mV in 5 mV increments) were applied every 5 s. Insets show persistent inward currents (normalized by maximal transient peak currents) from WT and p.Asn1768Asp channels at the end of a 100 ms step depolarization to −80 mV (black, control) and +20 mV (red). [reprinted from Veeramah et al. (2012), with permission].

The first *de novo* mutation in *SCN8A* was discovered in 2012 by whole genome sequencing of a child with an early onset, debilitating epileptic encephalopathy. The clinical picture included developmental delay, features of autism, intellectual disability and ataxia (Veeramah et al., 2012). Afebrile seizures began at 6 months of age, and by 5 years EEG recordings detected short bursts of frontocentrally predominant generalized spike-wave activity, and bifrontal and multifocal spikes. Neither the parents nor an unaffected sibling carried the *de novo* mutation, p.Asn1768Asp, that was detected in the patient. The biophysical properties of the mutant channel include increase in persistent sodium current, incomplete channel inactivation, and a depolarizing shift in the voltage dependence of steady-state fast-inactivation (Veeramah et al., 2012). Current tracings of cells transfected with mutant channels reveal as much as 20% of maximal current remaining after 100 ms, compared with only 1% in cells transfected with wild-type channel (Figure 1). The elevated persistent current increases the likelihood of premature firing of neurons after subthreshold depolarization. Transfection of mouse hippocampal neurons with the mutant cDNA resulted in increased spontaneous and induced firing characteristic of neuronal hyperexcitability, consistent with the dominant expression of seizures in the heterozygous patient. Increased persistent current is also a common feature of mutations in the channel *SCN1A* that cause the epileptic encephalopathy Dravet Syndrome (Meisler and Kearney, 2005). Increased activity of Na_v_1.6 has also been implicated in the seizure-prone *Celf4*^−/−^ mouse mutant (Sun et al., 2013) and suggested in fibroblast-derived neurons from patients with Dravet syndrome (Liu et al., 2013).

**Figure 2:**
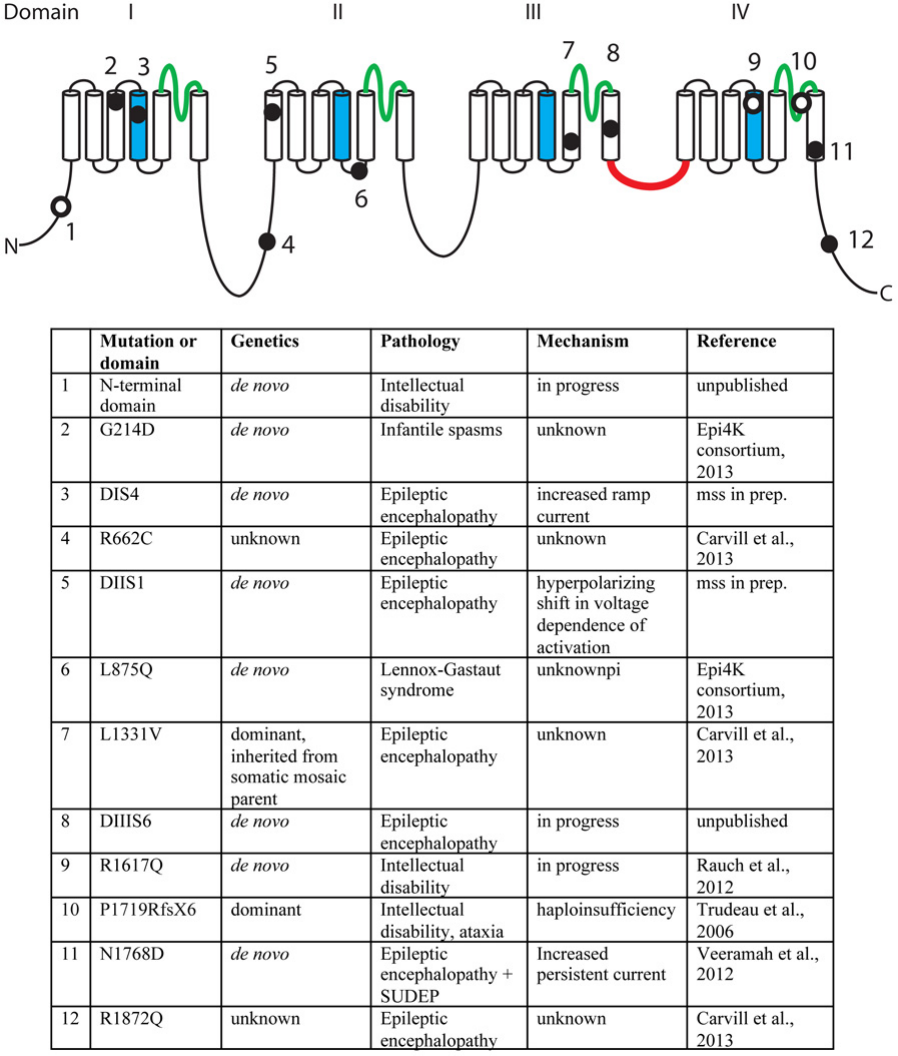
**Mutations of human *SCN8A*.** The positions of twelve recently identified mutations of *SCN8A* are indicated on the backbone of the channel structure. The four homologous domains are labeled with the pore domains in green, the voltage-sensing transmembrane segments (S4) in blue, and the inactivation gate in red. Filled circles, mutations identified in patients with epilepsy. Open circles, mutations identified in patients with cognitive deficits. Unpublished mutations are shown in their approximate positions.

A second missense mutation, *SCN8A*-p.Leu1331Val, was identified by targeted resequencing of 65 candidate genes in 500 individuals with epileptic encephalopathy (Carvill et al., 2013). The proband presented with epileptic encephalopathy at 18 months of age, and the mutation was inherited from a mosaic father. Two additional mutations were identified in this study, p.Arg662Cys and p.Arg1872Gln, but family data regarding inheritance was not available (Carvill et al., 2013). The mutation, *SCN8A*-p.Arg223Gly, was recently identified in child that presented with epileptic encephalopathy at 6 months of age (Kovel et al., submitted). In a screen for *de novo* mutations in 264 patients with infantile spasms or Lennox-Gastaut syndrome, the *SCN8A* mutation p.Leu876Gln was found in a child with Lennox-Gastaut (Epi4K Consortium and Epilepsy Phenome/Genome Project, 2013). The locations of the epilepsy-associated mutations are indicated in Figure 2.

## Mutations of *SCN8A* in intellectual disability

In 2006, we described the heterozygous loss-of-function mutation P1719RfsX1724 that segregated with cognitive deficits in a small family (Trudeau et al., 2006). Heterozygous children in this family were enrolled in special education classes, and heterozygous adults were unable to live independently. In 2012, Rauch and colleagues sequenced the exomes of 51 individuals with severe non-syndromic intellectual disability (Rauch et al., 2012). These patients were offspring of healthy, non-consanguineous parents and presented with intellectual disability, grossly normal motor function, and lack of syndrome-specific abnormality. The *de novo* missense variant p.Arg1617Gln in the voltage-sensing transmembrane segment of domain 4 of *SCN8A* was identified in one patient (Figure 2). Four additional *de novo* missense mutations in *SCN8A* have been discovered by exome sequencing of patients with intellectual disability (Figure 2). The limited functional data suggest that mutations causing increased channel activity are associated with seizures, while heterozygous loss-of-function of *SCN8A* predisposes to intellectual disability (Figure 2).

## Mutations of *Scn8a* in the mouse

**Figure 3:**
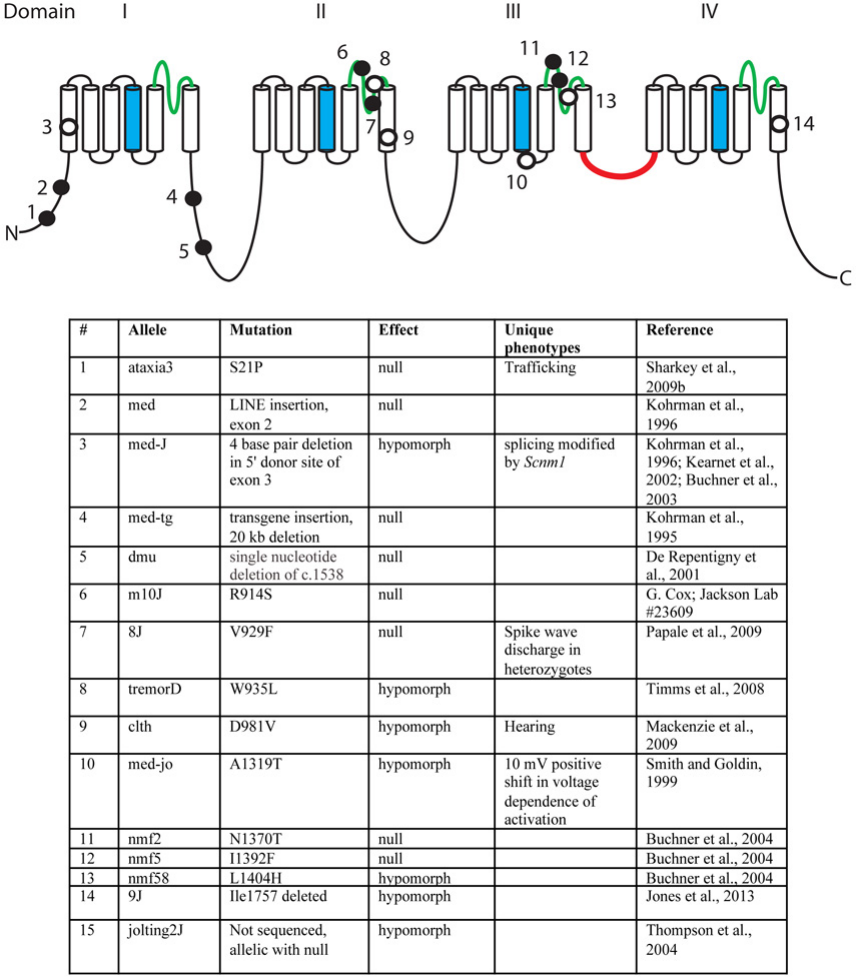
**Mutations of mouse *Scn8a*.** Fourteen allelic mutations are shown on the channel backbone as described in Figure 2. Amino acids are numbered according to Genbank AF049617. Filled circles, null alleles; open circles, hypomorphic alleles.

Over the past 18 years, fifteen mutant alleles of mouse *Scn8a* have been characterized. These include six spontaneous mutants, eight ENU-induced mutations, and one random transgene insertion (Figure 3) (Meisler et al., 2004). Several of these are null mutations with complete loss of *Scn8a* function. Homozygous null mice exhibit motor defects at 2 weeks of age, including ataxia and tremor, and do not survive beyond 3 weeks (Burgess et al., 1995; Kohrman et al., 1995). Homozygosity for severe hypomorphic alleles such as *medJ* and *nmf58* is viable, but results in ataxia and tremor with progression to muscle weakness and dystonia. Homozygosity for five mildly hypomorphic alleles (*medjo, jolting2J, tremorD, clth, 9J*) results in tremor, ataxia and reduced body size. These observations suggest that mutations of human *SCN8A* may be found in the future in patients with movement disorders. Na_v_1.6 is expressed at a low level in cardiac myocytes, and null mice have prolonged cardiac action potentials, suggesting a possible role in cardiac arrythmias (Noujaim et al., 2012). Homozygous knockout of *Scn8a* in Purkinje cells results in impaired learning in Morris Water Maze and eyeblink conditioning tests (Woodruff-Pak et al., 2006).

Mice that are *heterozygous* for loss-of-function mutations exhibit minor abnormalities such as spike-wave discharges suggestive of absence epilepsy (Papale et al., 2009), disrupted sleep architecture (Papale et al., 2010), and behavioral deficits including anxiety (McKinney et al., 2008). Haploinsufficiency of Na_v_1.6 also reduces susceptibility to genetic- and chemically-induced seizures (Martin et al., 2007, 2010). *Scn8a*^*med/*^^+^ and *Scn8a*^*med−jo/*^^+^ heterozygotes have reduced susceptibility to flurothyl and kainic acid induced seizures, and the combination of one mutant allele of *Scn8a* with Na_v_1.1 heterozygous or homozygous null mice results in extended lifespan and reduced seizure susceptibility. These observations suggest that reduced expression of *Scn8a* protects against seizures by decreasing neuronal excitability.

## Loss of Na_v_1.6 reduces neuronal excitability in mutant mice

Direct evidence for the *in vivo* role of Na_v_1.6 has been advanced by recordings from neurons from several different lines of *Scn8a* null and conditional null mice developed in our laboratory (Burgess et al., 1995; Levin and Meisler, 2004; Levin et al., 2006) (Table 1). Reduced repetitive firing is consistently observed in cerebellar Purkinje cells, granule neurons, trigeminal mesencephalic neurons, and retinal ganglion cells from *Scn8a* mutant mice (Raman and Bean, 1997; Raman et al., 1997; Van Wart and Matthews, 2006; Aman and Raman, 2007). Reduced persistent and resurgent current was observed in several types of neurons by multiple investigators (Table 1). In addition to induced firing, spontaneous firing is reduced in Purkinje neurons isolated from null mice (Khaliq et al., 2003). Overall, the work summarized in Table 1 demonstrates that *Scn8a* is a key determinant of neuronal excitability *in vivo*.

**Table 1:** **Reduced activity of neurons from *Scn8a* null mice**.

	**Neuron**	**Mutant mouse**	**Neuronal activity**	**References**
1	Cerebellar Purkinje cells	*med-tg, med*	Reduced repetitive firing, reduced resurgent current (−70%), reduced transient current (−50%)	Raman et al., 1997; Aman and Raman, 2007
2	Cerebellar granule cells	*Conditional knockout*	Reduced persistent current, reduced firing rate	Osorio et al., 2010
3	Trigeminal-mesencephalic	*med*	Reduced repetitive firing reduced resurgent current (−40%), reduced persistent current (−75%),	Enomoto et al., 2007
4	Retinal ganglion	*med-tg*	Reduced repetitive firing	Van Wart and Matthews, 2006
5	Cerebellar nucleus	*med*	No significant changes	Aman and Raman, 2007
6	DRG large and small diameter	*med-tg*	Reduced resurgent current (−100%)	Cummins et al., 2005
7	Subthallamic	*med*	Reduced resurgent current, altered firing	Do and Bean, 2004
8	Prefrontal cortical pyramidal	*med-tg*	Reduced resurgent current	Maurice et al., 2001
9	Hippocampal CA1	*med* (Stock No. 003798)	Reduced persistent, reduced resurgent, significant elevation of spike threshold, altered spike initiation, reduced spike gain	Royeck et al., 2008
10	Motor neurons	*med-J*	Reduced conduction velocity	Kearney et al., 2002
11	Globus pallidus neurons	*med-tg*	Impaired pacemaking, impaired capacity for fast spiking	Mercer et al., 2007

## Unique biophysical properties of Na_v_1.6

The role of *Scn8a* in regulating neuronal excitability may be related to three properties of Na_v_1.6: its role in persistent and resurgent current, its voltage dependence of activation, and its subcellular localization at the axon initial segment (AIS), the site of initiation of action potentials. Persistent current is a steady-state sodium current that persists after firing and is involved in action potential initiation at membrane voltages near the threshold of firing (Crill, 1996; Smith et al., 1998; Rush et al., 2005; Osorio et al., 2010). Persistent current is important for generation of repetitive firing in neurons such as cerebellar Purkinje cells. In cerebellar Purkinje cells isolated from *Scn8a* null mice, persistent current was reduced by 70% compared with wild-type littermates (Raman et al., 1997). In tsA-201 kidney cells, the persistent current generated by Na_v_1.6 is five-fold higher than that generated by Na_v_1.2 (Chen et al., 2008). The differences in magnitude of persistent current in different types of neurons suggests that this property is modulated by neuron-specific factors (Rush et al., 2005; Chen et al., 2008). Mutations that further increase Na_v_1.6 persistent current result in epileptogenesis (e.g., Figure 1) (Veeramah et al., 2012).

Resurgent current is a voltage- and time-dependent property in which depolarization after the initial action potential elicits a small, transient current (Hille, 2001). This rapidly reversible form of inactivation allows neurons to fire quickly and repetitively. Resurgent current is thought to contribute to spontaneous firing and multi-peaked action potentials in cerebellar Purkinje cells that are compromised in mutants lacking Na_v_1.6 (Raman and Bean, 1997; Raman et al., 1997). The β4 sodium channel subunit is involved in generating resurgent current in cerebellar Purkinje neurons and cerebellar granule cell neurons, but the blocking factor appears to vary by neuron type (Raman and Bean, 2001; Grieco et al., 2005; Bant and Raman, 2010).

In transfected DRG neurons, there is a 15 mV leftward shift in voltage dependence of fast activation of Na_v_1.6 compared to Na_v_1.2, meaning that Na_v_1.6 is more activated earlier during depolarization (Rush et al., 2005). Na_v_1.6 is also less likely to inactivate at higher stimulation frequencies (20–100 Hz) (Rush et al., 2005). In transfected HEK-tsA-201 cells, Na_v_1.6 displayed a more positive voltage dependence of slow inactivation, passing ~10% more current in the −35 to −25 mV range than Na_v_1.2 (Chen et al., 2008). These features of Na_v_1.6 contribute to the positive effect of Na_v_1.6 on neuronal excitability.

## Na_v_1.6 in the axon initial segment

The AIS is the membrane domain at the proximal end of the axon in which sodium channels are highly concentrated, electrical signals from the soma and dendrites are summed, and the threshold for action potential initiation is lowest (Royeck et al., 2008). The channel composition of the AIS appears to determine the firing threshold for different types of neurons (Lorincz and Nusser, 2008). Na_v_1.6 is highly concentrated in the distal half of the AIS in many neurons, including cerebellar granule cells and cerebellar Purkinje cells (Van Wart and Matthews, 2006; Lorincz and Nusser, 2008; Royeck et al., 2008). In the absence of Na_v_1.6, there is relocation of Na_v_1.1 and Na_v_1.2 to occupy the distal AIS (Van Wart and Matthews, 2006; Xiao et al., 2013). Cultured hippocampal CA1 pyramidal cells from *Scn8a*-null mice exhibit a 5 mV depolarizing (rightward) shift in the voltage dependence of activation, 60% reduction in persistent current, and 75% reduction in resurgent current (Royeck et al., 2008). This combination renders *Scn8a* null neurons less excitable than their wild type counterparts, as demonstrated by an 8 mV depolarizing shift in the spike threshold (Royeck et al., 2008).

In cortical pyramidal neurons, action potentials initiate at the distal part of the AIS, where sodium channel concentrations are highest (Van Wart et al., 2007; Kole and Stuart, 2008; Kole et al., 2008). The distal AIS in these cells contains predominantly Na_v_1.6, while the proximal AIS contains predominantly Na_v_1.2 (Hu et al., 2009). Step-depolarizations of patched neurons revealed that the activation threshold in the distal AIS was −55 mV, while the activation threshold in the proximal AIS closest to the soma was −43 mV (Hu et al., 2009), consistent with a role for Na_v_1.6 in lowering the threshold of action potential initiation.

Action potentials are primarily directed down the axon, away from the soma, but backpropagation into the soma occurs at low frequency (Hu et al., 2009). Current injection into the distal AIS does not produce backpropagation, while current injection at the proximal AIS leads to detectable action potentials in the soma (Hu et al., 2009). Thus, localization of Na_v_1.6 to the distal AIS is associated with a lower threshold for action potential initiation and direction of the action potential away from the soma. Overall, membranes containing Na_v_1.6 are more excitable than those containing only Na_v_1.1 and Na_v_1.2, and loss of Na_v_1.6 results in a higher threshold for initiation of action potentials (Van Wart and Matthews, 2006).

## Molecular features of *SCN8A*

The *SCN8A* gene is located on human chromosome 12q13.13 (Plummer et al., 1998) and mouse distal chromosome 15 (Burgess et al., 1995). The 27 exons of *SCN8A* span 170 kb and encode a protein of 1980 residues (GenBank AF050736). The location of the amino acid residues within the 4 homologous domains and 24 transmembrane segments of Na_v_1.6 is shown in Figure S1. Na_v_1.6 protein is concentrated ~1,000-fold in two membrane domains, the AIS and the nodes of Ranvier of myelinated axons (Schaller and Caldwell, 2000; Boiko et al., 2001, 2003; Van Wart and Matthews, 2006; Van Wart et al., 2007; Lorincz and Nusser, 2008, 2010). Na_v_1.6 is also present at lower abundance in non-myelinated axons, neuronal soma, and dendrites (Krzemien et al., 2000; Lorincz and Nusser, 2010). The full-length *SCN8A* transcript is highly expressed throughout the brain, with concentration in the cerebellum and olfactory bulb of the rat (Schaller and Caldwell, 2000).

Transcriptional regulation of sodium channel genes is not well characterized. The transcription start sites for *Scn8a* are located in noncoding exons 70 kb upstream of the translation initiation site (Drews et al., 2005). Exon 1c is highly conserved through evolution and includes potential binding sites for neuronal transcription factors Pou6f1/Brn5, YY1, and REST/NRSF (Drews et al., 2007). Exon 1c and upstream sequences are sufficient to drive neuron-specific expression of LacZ in transgenic mice (Drews et al., 2007).

*SCN8A* contains two pairs of mutually exclusive, alternative coding exons whose splicing regulates channel function. Exons 5N/5A and 18N/18A encode the S3–S4 transmembrane segments of domain I and domain III, respectively (Plummer et al., 1997). Exon 18N contains an in-frame stop codon and is only expressed in non-neuronal cells (Plummer et al., 1997) including glia (O'Brien et al., 2012a). The neuronal splice factors RBFOX1 and RBFOX2 can activate inclusion of exon 18A in neurons, resulting in neuron-specific expression of the full length, active channel (Gehman et al., 2012; O'Brien et al., 2012a; Zubovic et al., 2012). Splice enhancers and silencers in exons 18A and 18N also contribute to temporal and spatial regulation (Zubovic et al., 2012). Alternative polyadenylation sites are located 4 and 6.5 kb downstream from the translation termination site of *Scn8a*, generating full-length coding transcripts of 9 and 12 kb (Drews et al., 2005). Transcripts with the shorter and longer 3′ UTR are equally represented in brain RNA and are not known to be associated with specific functions.

## Pharmacology of Na_v_1.6

The pharmacology of compounds that target voltage-gated sodium channels has recently been reviewed (Eijkelkamp et al., 2012). The epileptic encephalopathies described in this review could in principle be treated with specific inhibitors of Na_v_1.6. However, the extensive sequence conservation among the neuronal and muscle sodium channels has made it difficult to develop drugs with specificity for a single channel. Two compounds with preferential effects on Na_v_1.6 have been described. The tetrodotoxin derivative 4,9-anhydrotetrodotoxin inactivates Na_v_1.6 expressed in Xenopus oocytes at concentrations that have minimal effects on six of the other channels (Rosker et al., 2007). The beta-scorpion toxin Cn2 also binds Na_v_1.6 specifically (Schiavon et al., 2006); this compound enhanced resurgent current inducing a hyperpolarizing shift in voltage dependence of channel activation in Purkinje slices, indicative of channel activation, while in HEK cells the effect was inhibitory. We have generated a mouse model of epileptic encephalopathy carrying the *SCN8A*-p.Asn1768Asp mutation that may be useful for future evaluation of drug specificity and effectiveness *in vivo*.

## Protein interactions of Na_v_1.6

**Figure 4:**
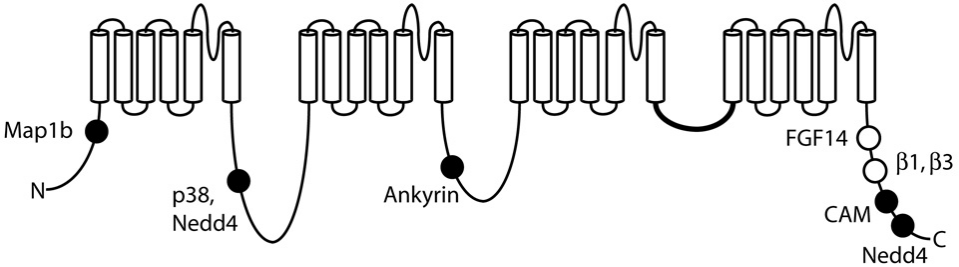
**Locations of protein interactions with Na_v_1.6.** Filled circles represent binding sites that have been localized to specific residues of Na_v_1.6: Map1b (77–80), p38 (553), ankyrin (1089–1122), calmodulin (1902–1912), and Nedd4 (551–554 and 1943–1945). Open symbols, binding sites that have not been mapped to specific residues.

Voltage-gated sodium channels are components of large, multi-protein complexes that vary between neurons and at specific subcellular domains. The known sites of protein interaction with Na_v_1.6 are indicated in Figure 4. The N-terminus of Na_v_1.6 interacts with the light chain of microtubule-associated protein Map1b (*Mtap1b*), and co-transfection increases current density in transfected cells via increased trafficking of Na_v_1.6 to the cell surface (O'Brien et al., 2012b). Phosphorylation of Na_v_1.6 by the stress-activated MAP kinase p38 facilitates binding of E3 ubiquitin ligases and channel degradation (Sudol and Hunter, 2000; Zarrinpar and Lim, 2000; Gasser et al., 2010). Protein kinases PKA and PKC have only a small effect on channel activity (Chen et al., 2008). Ankyrin G binds to the first intracellular loop of Na_v_1.6 and other neuronal sodium channels (Srinivasan et al., 1988; Davis et al., 1996; Hill et al., 2008), and is essential for targeting and localization of Na_v_1.6 to nodes of Ranvier (Gasser et al., 2012).

The intracellular fibroblast growth factors FGF11-FGF14 interact with Na_v_1.6 and other voltage-gated sodium channels (Wittmack et al., 2004; Laezza et al., 2009; Shakkottai et al., 2009; Xiao et al., 2013). FGF13 interacts with the C-terminus in an isoform-dependent manner (Wittmack et al., 2004), which may allow specific sub-populations of neurons to fine-tune firing properties via alternative splicing of FGF13. *Fgf14* null mice develop ataxia and ~80% of their cerebellar Purkinje cells lack repetitive firing (Shakkottai et al., 2009). The abundance of Na_v_1.6 in the AIS is reduced in cerebellar Purkinje cells from *Fgf14* null mice, suggesting that *FGF14* plays a key role in the organization of a subunits in the AIS (Xiao et al., 2013).

The sodium channel subunits β1 to β4 are small single-transmembrane cell-adhesion molecule proteins that modulate current and surface expression of the α subunit (Patino and Isom, 2010). Studies of mice null for the β1 subunit (*Scn1b*^−/−^) suggest that interaction between β1 and Na_v_1.6 is required for function of Na_v_1.6 at the distal AIS (Brackenbury et al., 2010). The β4 subunit has been implicated in the generation of resurgent Na_v_1.6 current in Purkinje neurons (Grieco et al., 2005; Aman et al., 2009), but resurgent current was not generated by co-transfection of β4 and Na_v_1.6 in HEK cells (Chen et al., 2008; Aman et al., 2009).

The calcium responsive protein calmodulin binds the IQ motif located in the C-terminus of Na_v_1.6 (residues 1902–1912). Apo-calmodulin accelerates inactivation and Ca^2+^ increases excitability of Na_v_1.6 (Herzog et al., 2003). The E3 ubiquitin ligase Nedd4 also binds to the C-terminus of *Scn8a* at a PXY motif (residues 1943–1945), and the PXpS/pTP motif in the first cytoplasmic loop (residues 551–554) (Abriel et al., 2000; Sudol and Hunter, 2000; Fotia et al., 2004; Ingham et al., 2004; van Bemmelen et al., 2004; Rougier et al., 2005). Both sites are necessary for Nedd4 binding and internalization of Na_v_1.6 (Gasser et al., 2010). Ubiquitination of Na_v_1.6 by Nedd4 is thought to target Na_v_1.6 for degradation and may be part of the neuronal stress response.

These interactions are relevant to the genetics of neurological and psychiatric disorders, since proteins that bind Na_v_1.6 may be considered candidate genes for the same disorders caused by mutations of Na_v_1.6. Further, common variants of the interacting proteins may act as modifiers of the severity of *SCN8A* mutations in patients (Meisler et al., 2010; Meisler and O'Brien, 2012).

## Conclusion

Na_v_1.6 is a major sodium channel in human brain. The features of Na_v_1.6 that influence neuronal excitability include contributions to persistent and resurgent neuronal currents, low threshold for excitation, and concentration in the AIS. Mutations of *Scn8a* in the mouse result in movement disorders including ataxia, dystonia, and tremor. Within the past year, *de novo* mutations of human *SCN8A* detected by exome sequencing have revealed a role for Na_v_1.6 in epilepsy and intellectual disability. Hypoactivity and hyperactivity of Na_v_1.6 are both pathogenic, but with different outcomes: haploinsufficiency is associated with impaired cognition (Trudeau et al., 2006; McKinney et al., 2008; Rauch et al., 2012) while hyperactivity can result in epilepsy (Veeramah et al., 2012). Analysis of additional mutants in the near future should provide insight into structure-function relationships of Na_v_1.6 and the mechanisms of pathogenesis in neurological disease.

### Conflict of interest statement

The authors declare that the research was conducted in the absence of any commercial or financial relationships that could be construed as a potential conflict of interest.

## Supplementary material

Figure S1 **Amino acid sequence of human and mouse *SCN8A***. The predicted human amino acid sequence corresponds to the predominant transcript in adult brain containing exon 5A and exon 18A and using the upstream splice donor site of exon 10B. The approximate locations of transmembrane segments S1–S6 of domains I–IV of the protein are underlined. Human coding sequence, GenBank AF050736; mouse coding sequence, GenBank AF049617. ∇, exon borders; ^*^, protein kinase A consensus sequence; arrow, tyrosine kinase consensus; dots, amino acid identity. Adapted from Plummer et al. (1998).
