# Supplementary material for: Sodium channel SCN8A (Nav1.6): properties and de novo mutations in epileptic encephalopathy and intellectual disability
Source: Front Genet. 2013 Oct 28;4:213. doi: 10.3389/fgene.2013.00213 (PMC3809569; doi:10.3389/fgene.2013.00213)
Supplement: Figure S1 — Amino acid sequence of human and mouse SCN8A. The predicted human amino acid sequence corresponds to the predominant transcript in adult brain containing exon 5A and exon 18A and using the upstream splice donor site of exon 10B. The approximate locations of transmembrane segments S1–S6 of domains I–IV of the protein are underlined. Human coding sequence, GenBank AF050736; mouse coding sequence, GenBank AF049617. ∇, exon borders; *, protein kinase A consensus sequence; arrow, tyrosine kinase consensus; dots, amino acid identity. Adapted from Plummer et al. (1998). [file Presentation1.PDF]

```
Human MAARLLAPPG PDSFKPFTPE SLANIERRIA ESKLKKPPKA DGSHREDDED SKPKPNSDLE AGKSLPFIYG DIPQGLVAVP LEDFDPYYLT 90
Mouse ....V..... .......... .......... .......... .......... .......... .......... .......... ..........

      1 ▽ 2                                      2 ▽ 3                            3 ▽ 4
      QKTFVVLNRG KTLFRFSATP ALYILSPFNL IRRIAIKILI HSVFSMIIMC TILTNCVFMT FSNPPDWSKN VEYTFTGIYT FESLVKIIAR 180
      .......... .......... .......... .......... .......... .......... .....E.... .......... ..........
                                                          IS1                         IS2

                                  4 ▽ 5A                       5A ▽ 6
      GFCIDGFTFL RDPWNWLDFS VIMMAYVTEF VDLGNVSALR TFRVLRALKT ISVIPGLKTI VGALIQSVKK LSDVMILTVF CLSVFALIGL 270
      .......... .......... .......... .......... .......... .......... .......... .......... ..........
                            IS3                    IS4                                          IS5

                                               6 ▽ 7                  7 ▽ 8
      QLFMGNLRNK CVVWPINFNE SYLENGTKGF DWEEYINNKT NFYTVPGMLE PLLCGNSSDA GQCPEGYQCM KAGRNPNYGY TSFDTFSWAF 360
      .......... .......... .......R.. .......... ...M...... .......... .......... .......... ..........

                       8 ▽ 9                                                                     9 ▽ 10A
      LALFRLMTQD YWENLYQLTL RAAGKTYMIF FVLVIFVGSF YLVNLILAVV AMAYEEQNQA TLEEAEQKEA EFKAMLEQLK KQQEEAQAAA 450
      .......... .......... .......... .......... .......... .......... .......... .......... ..........
                                           IS6

      MATSAGTVSE DAIEEEGEEG GGSPRSSSEI SKLSSKSAKE RRNRRKKRKQ KELSEGEEKG DPEKVFKSES EDGMRRKAFR LPDNRIGRKF 540
      .......... ........D. V........L .......... .......... .......... .......... .......... ..........

      10A ▽ 10B                                                       *                     *
      SIMNQSLLSI PGSPFLSRHN SKSSIFSFRG PGRFRDPGSE NEFADDEHST VEESEGRRDS LFIPIRARER RSSYSGYSGY SQGSRSSRIF 630
      .......... .......... .......... .......... .......... .......... .......... .......... ..C.......

                  *                     10B ▽ 10C                                    10C ▽ 11
      PSLRRSVKRN STVDCNGVVS LIGGPGSHIG GRLLPEATTE VEIKKKGPGS LLVSMDQLAS YGRKDRINSI MSVVTNTLVE ELEESQRKCP 720
      .......... .......... ..-......- .......... .......... .....E.... .......... .......... ..........

                                                                               11 ▽ 12
      PCWYKFANTF LIWECHPYWI KLKEIVNLIV MDPFVDLAIT ICIVLNTLFM AMEHHPMTPQ FEHVLAVGNL VFTGIFTAEM FLKLIAMDPY 810
      .......... .......... .......... .......... .......... .......... .......... .......... ..........
                                         IIS1                                  IIS2

                                        12 ▽ 13
      YYFQEGWNIF DGFIVSLSLM ELSLADVEGL SVLRSFRLLR VFKLAKSWPT LNMLIKIIGN SVGALGNLTL VLAIIVFIFA VVGMQLFGKS 900
      .......... .......... ..G....... .......... .......... .......... .......... .......... ..........
                    IIS3                     IIS4                                      IIS5

                                                                     13 ▽ 14
      YKECVCKINQ DCELPRWHMH DFFHSFLIVF RVLCGEWIET MWDCMEVAGQ AMCLIVFMMV MVIGNLVVLN LFLALLLSSF SADNLAATDD 990
      ........S. E.K......N .......... .......... .......... .......... .......... .......... ..........
                                                                        IIS6

      DGEMNNLQIS VIRIKKGVAW TKLKVHAFMQ AHFKQREADE VKPLDELYEK KANCIANHTG ADIHRNGDFQ KNGNGTTSGI GSSVEKYIID 1080
      .......... .......... A.V....... .......... .......... .......... V......... .......... ..........

                                                 14 ▽ 15                                    ↓      15 ▽ 16
      EDHMSFINNP NLTVRVPIAV GESDFENLNT EDVSSESDPE GSKDKLDDTS SSEGSTIDIK PEVEEVPVEQ PEEYLDPDAC FTEGCVQRFK 1170
      .......... .......... .......... .......... .......... .......... .......... .......... ..........

                                               16 ▽ 17
      CCQVNIEEGL GKSWWILRKT CFLIVEHNWF ETFIIFMILL SSGALAFEDI YIEQRKTIRT ILEYADKVFT YIFILEMLLK WTAYGFVKFF 1260
      .......... .......... .......... .......... .......... .......... .......... .......... ..........
                                    IIIS1                                    IIIS2

                 17 ▽ 18A                                  18A ▽ 19
      TNAWCWLDFL IVAVSLVSLI ANALGYSELG AIKSLRTLRA LRPLRALSRF EGMRVVVNAL VGAIPSIMNV LLVCLIFWLI FSIMGVNLFA 1350
      .......... .......... .......... .......... .......... .......... .......... .......... ..........
               IIIS3                        IIIS4                                              IIIS5

                                                                   19 ▽ 20              20 ▽ 21
      GKYHYCFNET SEIRFEIEDV NNKTECEKLM EGNNTEIRWK NVKINFDNVG AGYLALLQVA TFKGWMDIMY AAVDSRKPDE QPKYEDNIYM 1440
      .......... .......DE. ....D..... .......... .......... .......... .......... .......... ..D..G....

                                     21 ▽ 22                           22 ▽ 23
      YIYFIIFIIF GSFFTLNLFI GVIIDNFNQQ KKKFGGQDIF MTEEQKKYYN AMKKLGSKKP QKPIPRPLNK IQGIVFDFIT QQAFDIVIMM 1530
      ....V..... .......... .......... .......... .......... .......... .......... ........V. ..........
              IIIS6                                                                                IVS1

                                                                              23 ▽ 24
      LICLNMVTMM VETDTQSKQM ENILYWINLV FVIFFTCECV LKMFALRHYY FTIGWNIFDF VVVILSIVGM FLADIIEKYF VSPTLFRVIR 1620
      .......... .......... .......... .......... .......... .......... .......... .......... ..........
                                       IVS2                             IVS3

      LARIGRILRL IKGAKGIRTL LFALMMSLPA LFNIGLLLFL VMFIFSIFGM SNFAYVKHEA GIDDMFNFET FGNSMICLFQ ITTSAGWDGL 1710
      .......... .......... .......... .......... .......... .......... .......... .......... ..........
           IVS4                                      IVS5

      LLPILNRPPD CSLDKEHPGS GFKGDCGNPS VGIFFFVSYI IISFLIVVNM YIAIILENFS VATEESADPL SEDDFETFYE IWEKFDPDAT 1800
      .......... .......... .......... .......... .......... .......... .......... .......... ..........
                                                     IVS6

      QFIEYCKLAD FADALEHPLR VPKPNTIELI AMDLPMVSGD RIHCLDILFA FTKRVLGDSG ELDILRQQME ERFVASNPSK VSYEPITTTL 1890
      .......... .......... .......... .......... .......... .......... .......... .......... ..........

      RRKQEEVSAV VLQRAYRGHL ARRGFICKKT TSNKLENGGT HREKKESTPS TASLPSYDSV TKPEKEKQQR AEEGRRERAK RQKEVRESKC 1980
      .......... .......... .......R.I .......... .......... .......... ...D...... .......... ..........
```

Supplemental Figure 1
